# Comparison between superficial femoral artery stenting and bypass surgery in severe lower-limb ischaemia: a retrospective study

**DOI:** 10.5830/CVJA-2014-074

**Published:** 2015

**Authors:** J Islam, JV Robbs

**Affiliations:** Department of Vascular Surgery, Grey’s Hospital, Nelson R Mandela School of Medicine, University of KwaZulu-Natal, Pietermaritzburg, South Africa; University of KwaZulu-Natal, and Entabeni Hospital, Durban, South Africa

**Keywords:** superficial femoral artery, stenting, bypass, severe leg ischaemia

## Abstract

**Background:**

Symptomatic femoro-popliteal disease is treated by bypass surgery or angioplasty with or without stenting. The aim of this study was to compare the results of stenting and bypass surgery with regard to limb salvage in patients with severe leg ischaemia.

**Methods:**

A total of 213 patients with femoro-popliteal disease presenting with severe claudication or critical limb ischaemia between January 2009 and December 2013 were evaluated; 118 patients (139 limbs) had stents placed and 95 patients (104 limbs) had bypass surgery. Most (60%) presented with critical limb ischaemia (rest pain 40%, tissue necrosis 20%), and the remainder with severe claudication. The treatment groups had matching risk factors.

**Results:**

The average age was 66 years and 73% were male. Tissue necrosis was found in 26% of the stent group and 12% of the bypass group (*p* = 0.009). In the stent group 26% had adjunctive procedures, compared to 16% in the bypass group (*p* = 0.138). During the one-year follow up, there were 30 stent occlusions (22%) and 18 graft occlusions (17%) (*p* = 0.42). There were 14 major amputations (10%) in the stent group, and 13 (13%) in the bypass group (*p* = 0.68). Limb salvage rate was 90% in the stent group, and 88% in the bypass group (*p* = 0.68). There were no peri-operative deaths in the stent group, but one in the bypass group (1%). One-year mortality rate was equal (8%) in both groups (*p* = 1.00).

**Conclusion:**

One-year outcome was comparable in both groups with regard to mortality, stent or graft patency and limb salvage rates.

## Abstract

Symptomatic superficial femoral artery (SFA) disease presenting either with severe claudication or critical limb ischaemia is treated with bypass surgery and traditionally has been the ‘goldstandard’ procedure. Surgical bypass using autogenous vein or prosthetic grafts as a conduit is well accepted and there are comparable patencies and limb salvage rates with either conduit.1

There have been considerable advances in the last two decades in percutaneous endovascular technology for the treatment of SFA disease. The techniques that have been developed include percutaneous balloon angioplasty and stenting, with variable results.2,3 Despite having three different options, namely surgical bypass, balloon angioplasty and stenting, none is superior to the other.

Although the five-year primary patency rate of femoropopliteal above-the-knee bypass with autogenous saphenous vein is 70%, this method of treatment is invasive with long incisions in the lower extremities and a peri-operative complication rate of 12%.4 Vascular surgeons have become more experienced with catheter-based technology and due to the minimal invasiveness of the procedure, both patients and vascular surgeons are increasingly attracted to endovascular procedures. Mwipatayi *et al.*5 and Nguyen *et al.*6 found stenting resulted in equivalent outcomes when compared to balloon angioplasty alone, but Laird *et al.*7 found that self-expanding nitinol stents were associated with better angiographic results and improved patency compared with balloon angioplasty alone.

Randomised, controlled trials comparing bypass surgery and balloon angioplasty alone generally showed similar outcomes in terms of amputation-free survival but in the short term, surgery was more expensive than angioplasty.8 Another study comparing surgical bypass with balloon angioplasty and stenting showed better primary patency for the stent group (67%) than the bypass group (49%) and there were higher re-intervention rates in the bypass group.9

Since there are conflicting data in the literature regarding the success of different methods of treatment of SFA disease and there is a lack of consensus guidelines on the optimum management of SFA disease, the aim of this study was to compare the results of stenting and surgical bypass in the local environment with regard to limb salvage rates in patients with severe leg ischaemia.

## Methods

Patients with superficial femoral artery (femoro-popliteal) disease presenting to a single practice, with severe claudication (crippling), preventing them from performing their daily activities, or critical limb ischaemia, admitted to Entabeni Hospital, Durban, South Africa between January 2009 and December 2013 were culled from a prospectively maintained database. Two hundred and thirteen (213) patients were evaluated; 118 patients (139 limbs) had had stents placed and 95 (104 limbs) had had bypass surgery. We did not include patients who had had balloon angioplasty alone.

Most of the patients (60%) presented with critical limb ischaemia and the remainder with crippling claudication. Among the patients with critical limb ischaemia, 40% presented with rest pain and the remainder with tissue necrosis (20%). Both treatment groups had similar risk factors. Follow up comprised clinical review at one month, six months and yearly thereafter.

## Stent treatment group

Due to the demand for minimally invasive procedures by patients, and the frequent presence of multiple co-morbidities in poor operative-risk patients, our practice has focused on an endovascular-first approach for most of the patients with less extensive lesions [TASC (Trans-Atlantic Inter-Society Consensus) IIA and B], reserving open surgical bypass for patients who had more extensive lesions (TASC IIC and D) or femoral artery origin disease.

All stenting was performed in a hybrid endovascular operating theatre with fixed imaging capabilities. In most cases, ipsilateral antegrade access was obtained with a 6-F sheath by percutaneous groin puncture. Distal run-off vessels were evaluated before crossing the lesions. All patients were given intravenous heparin (80 IU/kg).

Accurate measurement of lesion length and vessel diameter was obtained by calibration techniques. Lesions were crossed with a hydrophilic guide wire and an angled, tapered catheter, and the sub-intimal technique was used in some of the complete occlusive lesions.

All patients received a self-expanding uncovered nitinol stent from different manufacturers. More than one stent was used in some patients. All stents were ballooned post deployment. Post stent procedures, all patients received a loading dose of 300 mg of clopidogrel followed by 75 mg daily for four weeks, and were given aspirin and statin therapy on a long-term basis.

## Bypass treatment group

Most bypasses were from the common femoral artery to above-the-knee popliteal artery, using polytetrafluoroethylene (PTFE) grafts. Reversed autogenous saphenous vein grafts were used when a suitable vein was available. All bypass grafts had a distaflo cuff configuration with ring reinforcement. Post operatively, all patients continued with aspirin and statin therapy on a long-term basis.

## Statistical analysis

In the case of quantitative data, means and 95% confidence intervals (95% CI) were reported around sample estimates. Fisher’s exact test (two-tailed) and the *t*-test (two-tailed) were used for differences in proportions. A *p*-value of ≤ 0.05 was considered significant.

## Results

**Fig. 1.**
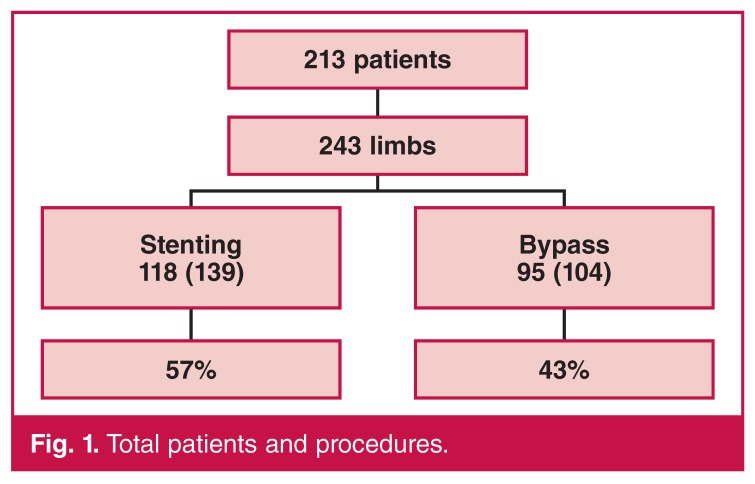
Total patients and procedures.

Two hundred and forty-three limbs were treated in 213 patients. Stenting was done in 139 limbs (57%) and bypass in 104 limbs (43%) (Fig. 1). The average age of the patients was 66 years (95% CI: 64.66–67.17), 73% were male and the male-to-female ratio was 2.73. The average age was similar in both treatment groups: 67 years (95% CI: 65.01–68.75) in the stent group and 65 years (95% CI: 63.17–66.27) in the bypass group (*p* = 0.08). The stent group had a similar gender distribution compared to the whole group (69% male and 31% female), whereas the bypass group had more males (79%), however this difference was not statistically significant (*p* = 0.11).

Critical limb ischaemia (CLI) was the presenting symptom in the majority of patients [128 (60%)]. Of these, 86 patients (40%) presented with rest pain and 42 (20%) with tissue necrosis or gangrene. The remainder of the patients presented with severe claudication [85 (40%)]. The distribution of severe claudication and critical limb ischaemia was similar in both treatment groups (Table 1), except that more patients presented with tissue necrosis in the stent group (26%) compared with the bypass group (12%) (*p* = 0.009).

**Table 1:** Clinical presentation

	*Stent n (%)*	*Bypass n (%)*	*p-value*
Severe claudication	42 (36)	43 (45)	0.16
Rest pain	45 (38)	41 (43)	0.48
Tissue necrosis	31 (26)	11 (12)	0.009
Total	118 (100)	95 (100)	

Percentage rounded to the nearest integer.

The prevalence of cardiovascular risk factors, for example hypertension, smoking, ischaemic heart disease (IHD), cerebrovascular disease (CVD), and renal failure was similar across the treatment groups, except for diabetes mellitus, which was higher in the stent group (51 vs 37%, *p* = 0.05), as shown in Table 2.. The presentations according to the TASC II classification are shown in Table 3.. Overall, 80% of TASC A and TASC B lesions received stents and 76% of TASC C and D lesions received bypass (*p* = 0.0001). In the stent group 26% of patients had adjunctive procedures, compared to 16% in the bypass group (*p* = 0.138) (Table 4).

**Table 2:** Demography and risk factors

	*Stent (n = 118) n (%)*	*Bypass (n = 95) n (%)*	*p-value*
Age (years)	67	65	0.08
Males	81 (69)	75 (79)	0.11
Hypertension	85 (72)	63 (66)	0.37
Diabetes	60 (51)	35 (37)	0.05
Smoking	65 (55)	51 (54)	0.89
IHD	45 (38)	30 (32)	0.38
CVD	12 (10)	12 (13)	0.66
Renal failure	9 (8)	6 (6)	0.79

IHD: ischaemic heart disease, CVD: cerebrovascular disease. Percentage rounded to the nearest integer.

**Table 3:** TASC II lesions

*TASC II*	*Number (%)*	*Stent (%)*	*Bypass (%)*
A	46 (19)	43 (93)	3 (7)
B	97 (40)	72 (74)	25 (26)
C	24 (10)	13 (54)	11 (46)
D	76 (31)	11 (14)	65 (86)
Total	243	139	104

TASC: Trans-Atlantic Inter-Society Consensus. Percentage rounded to the nearest integer.

**Table 4:** Adjunctive procedures

	*Stent*	*Bypass*	*p-value*
Popliteal-tibial angioplasty	22	0	
Iliac angioplasty/stenting	5	15	
CFA patch	4	0	
Total	31 (26%)	15 (16%)	0.13

CFA: common femoral artery.

During the one-year follow-up period there were 30 stent occlusions (22%). They were treated by balloon angioplasty alone (three patients), re-stenting (11), femoro-popliteal bypass (13), and three patients were treated conservatively. In the bypass group 18 patients had graft occlusions (17%) and they were managed by thrombectomy, angioplasty with or without stenting (five), or redo bypass (nine). Four septic grafts were removed.

There were 14 major amputations (10%) in the stent group and 13 (13%) in the bypass group (*p* = 0.68). The limb salvage rate was 90% in the stent group and 88% in the bypass group (*p* = 0.68). There were no peri-operative (30-day) deaths in the stent group, but one peri-operative death after discharge home due to an unknown cause in the bypass group (1%). One-year mortality rate was equal (8%) in both groups (*p* = 1.00), as shown in Table 5.. The causes of late deaths were myocardial infarction (10) and sepsis (two). In five patients the cause of death remained obscure.

**Table 5:** Outcomes after one year

	*Stent (n = 139) n (%)*	*Bypass (n = 104) n (%)*	*p-value*
Stent/graft occlusion	30 (22)	18 (17)	0.42
Major amputation	14 (10)	13 (13)	0.68
Death	10 (8)	8 (8)	1.00
Stent/graft patency rate	109 (78)	86 (83)	0.42
Limb salvage	125 (90)	91 (88)	0.68

Percentage rounded to the nearest integer.

## Discussion

All of our patients treated by endovascular techniques received a stent in addition to angioplasty. This is different from that reported in the BASIL (Bypass versus Angioplasty in Severe Ischaemia of the Leg) trial where patients in the endovascular group underwent angioplasty alone.8 During the same time period we had another 30 patients, who had balloon angioplasty done alone for the SFA disease, but we did not include them in this study, as we feel that balloon angioplasty and stenting have different results in terms of patency. This may differ from other authors.10

Stenting is appropriate for complex lesions and as a ‘bailout’ procedure after complications of balloon angioplasty, or recoil after balloon dilatation, and the outcome with stenting is superior to balloon angioplasty alone.6,7,11 We did not give preference to any specific stent, and a single stent was preferred in order to cover the entire lesion; multiple stents were however deployed if necessary.

In all cases, bare-metal, self-expanding nitinol stents were used. It has been reported that long-term outcomes of SFA intervention comparing endografts and bare-metal nitinol stents were similar.12 The average lesion length was 7.84 cm (range 4 to 20 cm) and the stent diameters ranged from 5 to 7 mm.

Eighty-six limbs (83%) had above-the-knee bypass, all using prosthetic grafts. Below-the-knee bypass was done in 18 (17%); a prosthetic graft was used in 14 of these and reversed saphenous vein grafts were used in four patients.

Although in one study the five-year patency and re-intervention rates were superior in above-the-knee bypass with saphenous vein grafts,13 other randomised, controlled trials showed that the outcome in terms of patency and limb salvage rates were comparable.1,4 Below-the-knee bypass with vein grafts is definitely superior to prosthetic grafts.14 Lack of availability of suitable veins precluded their use, and we were compelled to use prosthetic grafts in most cases.

Out of 86 above-the-knee prosthetic bypasses, 11 had a major amputation and of 18 below-the-knee bypasses, two resulted in amputation. Though no patient with a vein graft had an amputation, this was not statistically significant (*p* = 1.00).

The difference between stent and graft occlusion rates was not statistically significant (22 vs 17%, *p* = 0.42), and the stent and graft patency rates were similar in both groups: 109 (78%) in the stent group and 86 (83%) in the bypass group (*p* = 0.42). There was no difference in the major amputation rate between stents and bypasses, with 14 amputations (10%) in the stent group and 13 (13%) in the bypass group (*p* = 0.68). Among the patients who had amputation, 93% had presented with tissue necrosis in the stent group, and 46% in the bypass group (*p* = 0.01). The limb salvage rate was similar in both groups; 125 (90%) in the stent group and 91 (88%) in the bypass group (*p* = 0.68).

One-year mortality rate was similar in both groups; 10 (8%) in the stent group and eight (8%) in the bypass group (*p* = 1.00). The causes of late deaths were similar to previous reports, being mainly due to myocardial infarction. The current report was limited to a one-year follow up. This might have been responsible for the higher stent and graft patency, or limb salvage rates in comparison to other series.9,15 No patients were lost to follow up.

There are not many publications comparing femoral artery stenting and bypass surgery, and the assessment of patency and the overall results of different treatment modalities is somewhat problematic, as study designs vary considerably. 16-19 In our series 80% of TASC II A and B lesions received stents and 76% of TASC II C and D lesions received bypasses. These figures are in keeping with those reported in previous studies.9,18,19

There is evidence that shorter lesions do well with angioplasty/stent, while longer lesions have significantly lower patency rates.9 The latest TASC II recommendations include an endovascular approach for shorter lesions and a bypass for longer lesions.20 We followed this principle in our practice. Our study design is similar to that of Malas *et al.*9 and Linnakoski *et al.*21 and the outcomes are comparable. Notwithstanding we support the concept that an endovascular-first approach may be advisable in the elderly and in patients with significant co-morbidity.22,23

## Conclusion

Bearing in mind that this was a retrospective study, in the short term, stenting is a viable option to treat femoral artery occlusive disease. It is less invasive and equally effective compared to bypass surgery, especially in the elderly and in patients with high cardiovascular risk factors. One-year outcome was comparable in both groups with regard to mortality rate, stent or graft patency and limb salvage rates. There is a definite need for longterm follow up and a randomised, controlled trial to validate this.
